# Clinical course of poststroke epilepsy: a retrospective nested case–control study

**DOI:** 10.1002/brb3.366

**Published:** 2015-07-22

**Authors:** Johan Zelano, Rebecca Gertz Lundberg, Leopold Baars, Emelie Hedegärd, Eva Kumlien

**Affiliations:** Department of Neuroscience, Uppsala University751 85, Uppsala, Sweden

**Keywords:** Cerebrovascular diseases, epilepsy, treatment

## Abstract

**Introduction:**

Recently, several epidemiological studies have demonstrated that epilepsy develops after approximately 10% of all cerebrovascular lesions. With an aging population, poststroke epilepsy is likely to be of increasing relevance to neurologists and more knowledge on the condition is needed. Patients with poststroke epilepsy are likely to differ from other epilepsy patient populations regarding age, side-effect tolerability, comorbidities, and life expectancy, all of which are important aspects when counselling newly diagnosed patients to make informed treatment decisions.

**Method:**

We have here performed a nested case–control study on 36 patients with poststroke epilepsy and 55 controls that suffered stroke but did not develop epilepsy. The average follow-up time was between 3 and 4 years.

**Results:**

In our material, two-thirds of patients achieved seizure freedom and 25% experienced a prolonged seizure (status epilepticus) during the follow-up period. Cases consumed more health care following their stroke, but did not suffer more traumatic injuries. Interestingly, the mortality among cases and controls did not differ significantly. This observation needs to be confirmed in larger prospective studies, but indicate that poststroke epilepsy might not infer additional mortality in this patient group with considerable comorbidities.

**Conclusions:**

The observations presented can be of value in the counselling of patients, reducing the psychosocial impact of the diagnosis, and planning of future research on poststroke epilepsy.

## Introduction

Stroke is the leading cause of epilepsy in the elderly, accounting for approximately half of all newly diagnosed epilepsy cases above the age of 60 (Sander et al. 1990). Three recent studies have described the incidence of poststroke epilepsy in prospective follow-up studies, and found epilepsy rates after stroke of 8.2% (Jungehulsing et al. 2013), 11.3% (Arntz et al. 2013a), and 12.4%(Graham et al. 2013), after follow-up periods of 2, 9, and 10 years, respectively. With an aging population, poststroke epilepsy will most likely be of increasing interest to neurologists, especially since the importance of previous cerebrovascular lesions in assessing the risk of seizure recurrence has been encompassed in the new International League Against Epilepsy (ILAE) practical clinical definition of epilepsy (Fisher et al. 2014). The definition states that a single seizure can merit diagnosis of epilepsy if the recurrence rate is sufficiently high, for instance, if a stroke is present and the time frame for acute symptomatic seizure has passed. The new definition will most likely increase awareness of poststroke epilepsy, and as recognition of the condition increases, more information on the prognosis of the condition is needed for proper counselling of patients and informed treatment decisions.

The epidemiological studies cited above have shed considerable light on the risks of developing poststroke epilepsy after stroke. The most important risk factors are also known; cortical location, hemorrhagic stroke, and residual neurological symptoms (Strzelczyk et al. 2010). Nonetheless, the diagnosis of poststroke epilepsy can be challenging and therefore delayed, as illustrated by one study in which one-fifth of stroke mimics were in fact seizures (Hand et al. 2006). Hopefully, the increased knowledge on epidemiology, the updated ILAE definition, and awareness of diagnostic pitfalls will increase early recognition of patients suffering from poststroke epilepsy.

Efforts must now be directed at gathering more knowledge on the management of patients after diagnosis. The available data on risks of acquiring poststroke epilepsy is not matched by knowledge on the condition itself and currently there is a great scarcity of data to guide clinicians once a diagnosis has been made and the health care system needs information on how much additional resources are required when recovery from stroke is complicated by recurrent seizures. Reasonable questions posed by patients include the expected treatment response, the risk of status epilepticus or seizure-related injury, the amount of extra health care that will result from the epilepsy, and the impact on mortality. These issues have largely been ignored, with some exceptions. Arntz et al. (2013b) demonstrated that epilepsy contributes to detrimental outcome in young patients with stroke or transitory ischemic attack (TIA).

We here report a retrospective nested case–control study, in which we have attempted to capture the most important prognostic aspects of poststroke epilepsy. Cases are compared to controls that suffered stroke, but did not develop epilepsy. We first describe the epilepsy and epilepsy-specific prognosis; what percentage of patients will suffer status epilepticus, how many will achieve seizure freedom, and how much consumption of health care that will be related to epilepsy. Finally, we also made some observations on mortality and causes of death. Hopefully, this descriptive study will contribute to systematic knowledge that can guide future research and counselling of patients with poststroke epilepsy.

## Material and Methods

### Study population

**Figure 1:**
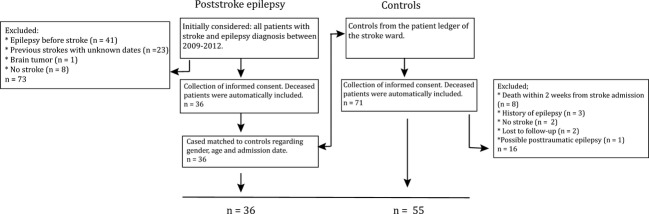
Study design. Flowchart over enrolment.

The study design was a nested case–control study. Patients with poststroke epilepsy were recruited from the all-encompassing electronic patient records system at Uppsala university hospital, a tertiary hospital serving a primary population of approximately 280,000 and a secondary population of 2 million (Fig.1). All patients with a diagnosis of stroke during 2009–2012 and a diagnosis of epilepsy (defined as unprovoked seizures occurring more than 1 week after the stroke) were selected and the medical records reviewed for exclusion criteria; epilepsy prior to the stroke, treatment with antiepileptic drugs, and patients that had subsequently moved from Uppsala county. Controls that died during the first 2 weeks after the stroke (*n* = 8) were also excluded, since one has to survive for some time in order to be diagnosed with poststroke epilepsy. Informed consent was collected from living patients. Together with deceased patients, we collected 35 cases. Controls were selected from the patient ledger of the hospital stroke ward for 2009–2012 (containing 2789 admissions). For each case, the three gender-matched patients closest in age and with an admission date within 7 days before or after the case admission date were selected. Together with already deceased individuals, we collected 56 controls. One control subsequently developed poststroke epilepsy and was transferred to the case group. The relevant ethical body approved the study. Written informed consent was collected from patients still alive. The ethics committee waived the need for informed consent for access to the medical records of deceased patients. Patient data were anonymized and de-identified prior to analysis.

### Data analysis

The medical records were examined and data extracted according to a predefined form. A few patients suffered multiple strokes during the study period. For such patients, the stroke preceding the diagnosis of epilepsy was used for analysis. For controls that had suffered multiple strokes, the first stroke was used for analysis. Acute symptomatic seizures occurring within 1 week from a stroke were not counted as first seizures. Seizure freedom was defined as no more seizures being noted in the medical records during the time in the study. We used *χ*^2^ tests to compare categorical variables between patients with and without epilepsy and Mann–Whitney *U*-test to analyze continuous variables. To assess the overall survival, a survival analysis of mortality was illustrated by a Kaplan–Meier curve and analyzed with the Gehan–Breslow–Wilcoxon and Mantel–Haenszel test. Time of survival was measured from the time point of first stroke. Time in study/survival time was calculated until 2014-07-15 or death. The statistical analysis was performed using GraphPad Prism 6 statistical Software (version 6.0; GraphPad Software Inc., San Diego, CA) with two tails for all tests and significance set at *P *<* *0.05.

## Results

We first analyzed the demographics and stroke characteristics of the population. Cases and controls were well matched regarding age, gender, and number of drugs on admission, the latter serving as a surrogate marker for overall morbidity (Table 2013a). Cases had more often suffered a hemorrhagic stroke than controls (31% vs. 15%). On average, the cases had a higher modified Rankin score (mRs) on discharge (score of 3.4 ± 1.6 vs. 2.1 ± 1.3), and a longer hospital stay (including geriatric or rehab ward) following their stroke (median of 36 days vs. 8 days). The cases were also on a greater number of drugs 1 year after the stroke (information on drugs was only available at 1 year poststroke for 97% of cases and 65% of controls). The mean observation time was 1750 days for cases and 1255 days for controls.

**Table 1:** Demographics and stroke characteristics. The epilepsy group and control group were well matched regarding age, gender, and number of drugs on admission. The patients in the epilepsy group had more often suffered hemorrhagic stroke, a higher modified Rankin score on discharge, a greater number of drugs 1 year poststroke, and a longer hospital stay following their stroke

	Cases	Controls	*P*-value
*N*	36	55	
Gender			
Male	22 (61%)	39 (71%)	
Female	14 (39%)	16 (29%)	
Age (mean ± SD)	70.7 ± 10.39	69.3 ± 11.71	
No of drugs (mean ± SD)			
Before stroke	4.3 ± 3.5	5.3 ± 4.4	0.4711 (ns)
1 year poststroke*	9.5 ± 3.9	7.3 ± 4.5	0.0220
Stroke type			
Hemorrhagic	11 (31%)	8 (15%)	
Ischemic	25 (69%)	47 (85%)	
Median hospital stay (range)	36 (3–285)	8 (1–96)	0.0057
Modified Rankin score	3.4 ± 1.6	2.1 ± 1.3	0.0020
Mean time in study (±SD)	1750 ± 889	1255 ± 570	0.0175

SD, standard deviation.

*P*-values from Mann–Whitney *U*-test.

* Information on drugs was only available at 1 year poststroke for 97% of cases and 65% of controls.

We next described the clinical characteristics of poststroke epilepsy in the study population (Table 2013b). The median time to epilepsy diagnosis was 283 days, but the interindividual variation was large, with the earliest diagnosis being made already 55 days after the stroke. A quarter of the cases experienced status epilepticus during their time in the study, and among these the SE frequency was 0.53 episodes per year. We also studied data on drugs prescribed. Out of the 35 cases that were started on an antiepileptic drug (AED), 46% became seizure free on the first AED, and 55% on the first or the second AED. The retention rate for the first AED tried was 67%. Out of the 11 patients that switched to a second AED, 64% (7/11) did so because of lack of efficacy, and 36% (4/11) because of side effects.

**Table 2:** Clinical characteristics of poststroke epilepsy in the study population. The median time to epilepsy diagnosis was 283 days. A quarter of the population experienced status epilepticus. Among patients that ever suffered SE, the mean frequency was 0.53 episodes per year. Out of the 35 patients that were started on AED, 46% became seizure free on the first AED, and 55% after trying a second AED

Clinical features of poststroke epilepsy	
Median latency to onset of epilepsy (min–max)	283 days (55–2386)
Patients ever experiencing status epilepticus	9 (25%)
Mean SE frequency/patient/year (min–max)	0.53 (0.18–0.93)
Seizure free after first AED (*n* = 35)	16 (46%)
Seizure free after first or second AED	20 (55%)
Remained on first AED (retention rate)	24 (67%)

SE, status epilepticus.

We next examined how much health care patients with poststroke epilepsy had consumed had been compared to controls (Table 2014). Since cases had generally suffered a more severe stroke, we stratified the cases and controls into two groups based on their stroke severity as assessed by the mRs on discharge. We decided on mRs of 2 as a divider, a level that indicates a need for assistance by another person for ambulation or daily activities. Nine cases and 32 controls had mRs of less than 2, with average mRs scores of 1.1 and 1.2 in the groups, respectively. Twenty-seven cases and 23 controls had mRs above 2, with average mRs scores in the groups of 4.1 and 3.2, respectively. In the less severely affected group, there was no significant difference in mRs score between cases and controls, but in the more severely affected group, the mRs score for cases was significantly higher than that of controls. Among cases and controls with mRs less than two, cases were significantly more often hospitalized (0.7 vs. 0.0 times/year, *P* = 0.001) and had a significantly higher frequency of emergency room (ER) visits (0.9 vs. 0.2 times/year, *P* = 0.0018) than controls. The same result was seen in the group of more severely affected patients (1.6 vs. 0.8 visits per year, *P* = 0.001, and 1.8 vs. 0.5 ER visits per year, *P* = 0.0006). The total time spent in hospital (days per year) showed a very large individual variation, and no significant difference could be detected between cases and controls in either group (data not shown). Traumatic events were not significantly more common in cases than controls.

**Table 3:** Health care consumption. Values are median (min–max). *P*-values from Kruskal–Walis test (multiple groups) or Mann–Whitney (two groups). Cases were significantly more often hospitalized and had significantly more ER visits than controls, both in the entire population and when stratified for stroke severity according to mRs. There was no significant difference in the frequency of traumatic injuries between the groups

Health care consumption Frequency (contacts per year [median (min–max)])			
All	Cases	Controls	*P*-value
Hospitalisations	1.4 (0.2–3.3)	0.3 (0.0–8.1)	<0.0001
Hospitalisations due to epilepsy	0.3 (0–2.8)		
Emergency visits	1.4 (0.1–4.6)	0.3 (0.0–10.0)	<0.0001
Emergency visits due to epilepsy	0.4 (0–2.1)		
Rankin ≤2	*n* = 9	*n* = 32	
Hospitalisations	0.7 (0.3–1.5)	0.0 (0.0–3.0)	0.0010
Hospitalisations due to epilepsy	0.2 (0–0.6)		
Emergency visits	0.9 (0.1–1.5)	0.2 (0.0–3.0)	0.0018
Emergency visits due to epilepsy	0.2 (0–0.5)		
Rankin >2	*n* = 27	*n* = 23	
Hospitalisations	1.6 (0.2–3.3)	0.8 (0.0–8.1)	0.0010
Hospitalisations due to epilepsy	0.5 (0–2.8)		
Emergency visits	1.8 (0.4–4.6)	0.5 (0.0–10.0)	0.0006
Emergency visits due to epilepsy	0.7 (0–2.1)		
Traumatic injuries (mean)			
Sutured wounds/years in study	0.058	0.019	0.0840
Number of fractures/years in study	0.055	0.050	0.3476

mRs, modified Rankin score.

**Figure 2:**
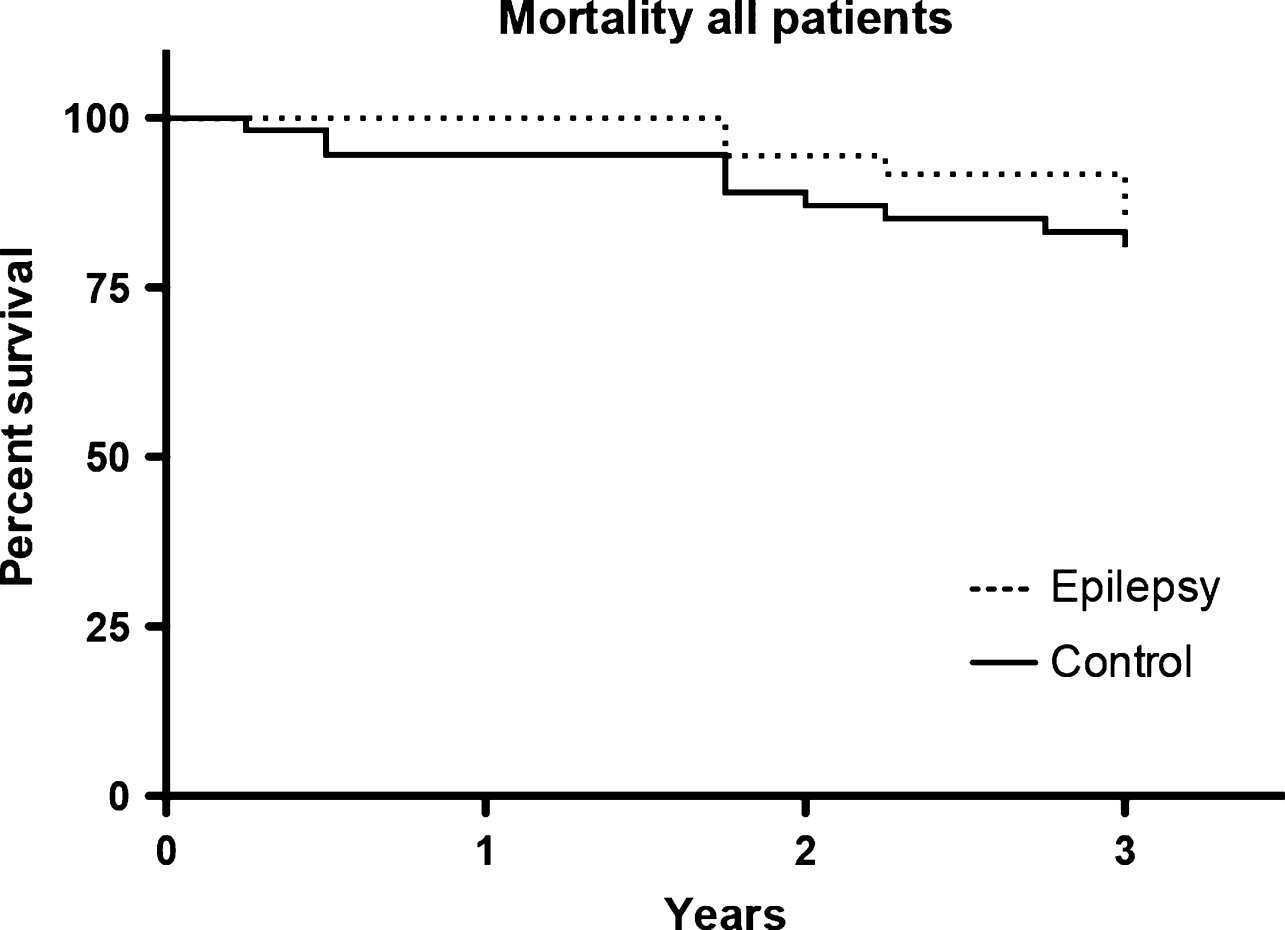
Kaplan–Meyer score of survival. Over the first 3 years in the study, no significant difference in mortality between the groups could be detected. All deaths occurred in subjects with a Rankin score above 2.

We finally turned to outcome. Causes of death were analyzed in all deceased patients, 15 patients and 15 controls. In both groups, a cardiovascular cause (stroke and cardiac causes) was listed in the majority of cases. Since the numbers were quite small no further statistical analysis was undertaken. No epilepsy-related cause of death was recorded in the records. Interestingly, there was no statistically significant difference between cases and controls when we assessed 3-year mortality (Fig.2). We also analyzed mortality with subjects stratified according to stroke severity. No deaths occurred in cases or controls with an mRs ≤ 2 and no significant difference in mortality between cases or controls with an mRs > 2 could be detected.

## Discussion

In this retrospective study, we have examined the prognosis of patients with poststroke epilepsy and compared it to a control group of stroke patients that did not develop epilepsy. To our knowledge, there are very few studies addressing the prognosis of this specific epilepsy population, which often present specific treatment challenges. Patients with poststroke epilepsy might have other neurological sequelae, cardiovascular comorbidities, or concomitant drugs, which are all factors that influence epilepsy care. For this reason, and because poststroke epilepsy is likely to become more common with an aging population, treatment of poststroke epilepsy should be based on disease-specific studies.

We identified 36 patients eligible for entry into the study. Importantly, the study was not designed to assess incidence or prevalence of poststroke epilepsy – but rather to select patients where we had optimal access to information on the clinical course. For instance, patients that relocated during the study time were excluded and the follow-up time was relatively short.

The time to diagnosis of poststroke epilepsy was highly variable in our cohort. The median time to diagnosis was similar to that observed in other populations (Strzelczyk et al. 2010), and the longer time spans seen in a few patients in our material might result from diagnostic delay rather than actual latency to the first seizure, since it is well recognized that seizures are often overlooked in the elderly population. Following diagnosis, all but one patient was started on AEDs, most commonly carbamazepine. The large proportion of patients started on carbamazepine was somewhat surprising and in our opinion patients diagnosed today are more likely to be prescribed alternative AEDs. It is possible that our results regarding discontinuation due to side effects reflect somewhat outdated prescription habits.

Importantly, 45% of patients achieved seizure freedom on the first AED. This is very similar to the rates of seizure freedom observed for lamotrigine and sustained-release carbamazepine in an international double-blind trial on treatment of new-onset epilepsy in the elderly (Saetre et al. 2007). Our data therefore give no reason for clinicians to have higher expectations or lower ambitions in the treatment of this patient group as a whole compared to other patients with epilepsy. However, 25% of the cases suffered an episode of status epilepticus during the study period, so it is possible that a subset of patients have difficulty to treat epilepsy.

We next compared cases to age- and gender-matched controls that suffered stroke at approximately the same time, but did not develop epilepsy. As expected, patients who developed epilepsy had suffered more severe strokes and more often hemorrhagic ones. This is hardly surprising, since stroke severity and stroke type are among the most important risk factors for poststroke epilepsy (Strzelczyk et al. 2010; Jungehulsing et al. 2013; Haapaniemi et al. 2014). Cases and controls were not different in baseline number of drugs on admission – a crude surrogate marker for overall morbidity – but 1 year after the stroke, cases were treated with more drugs. One might suspect that surplus agents include not only AEDs, but also cardiovascular risk factor modifiers and symptomatic treatment of other stroke sequelae, for instance spasticity.

Because of the differences in stroke severity, the ensuing analyses were performed with stratification for stroke severity, as measured by the modified Ranking score. Following diagnosis, cases consumed about twice as much health care as controls – and a large part of the difference was due to epilepsy-related visits or admissions. This difference was seen both in the total population and in the groups after stratification for stroke severity. Importantly, the number of ER visits should not be interpreted as a marker for seizure-related injuries, since previous reports have found a low incidence of seizure-related injuries in the elderly (Lawn et al. 2013). In our material, we also detected similar rates of fractures and wounds in cases and controls, indicating that poststroke epilepsy does not contribute significantly to traumatic injuries in this group.

Finally, we studied outcome. All deaths among study cases or controls were observed in subjects with mRs > 2. We were surprised that we could not detect any difference in mortality between the groups, this is, however, in keeping with the fact that no epilepsy-related causes of death were seen in the records. Importantly, Sweden has a low-autopsy frequency (none of the deceased patients underwent autopsy) and epilepsy-related deaths are difficult to detect in an elderly population. Deaths after seizures might be interpreted as cardiogenic or traumatic, but this hardly explains the similar frequency of cardiovascular deaths in controls. Our interpretation is that the results indicate (although weakly) that the poststroke epilepsy group might not contribute significantly to mortality in stroke patients with mRs > 2. Our follow-up time was most likely to short to assess mortality in patients with mRS < 2. Our study design does not allow generalization of epidemiological findings, but the impact of poststroke epilepsy on mortality needs to be studied further. If the observation in our study groups can be confirmed in future prospective studies, it might prevent poststroke epilepsy patients being attributed a worse prognosis than warranted and reduce the psychosocial impact of poststroke epilepsy.

The study has several limitations. First of all, the retrospective nature of the study makes it heavily reliant on the data entered into the medical records. We have tried to evaluate parameters that are somewhat insensitive to variations in clinician’s documentation habits – for instance, the number of outpatient visits rather than their content. However, one should emphasize that the results presented in this study are merely retrospective observations that should be confirmed in prospective investigations.

One can furthermore discuss our choice of controls. We found it interesting with a design that allows comparison with patients with stroke that did not develop subsequent epilepsy, since this is a reasonable comparator in discussions with patients. Nonetheless, one might argue that the control group is artificial since something fundamental must differ between cases and controls, or the latter would also have developed epilepsy. Indeed, our observations indicate that the controls suffered less severe strokes than cases and more often ischemic stroke. The controls were also less likely to have suffered previous strokes. We stratified the ensuing analyses according to time in study and stroke severity to compensate accordingly, but future and larger studies are needed to confirm our observations.

In summary, this is one of the first studies systematically describing poststroke epilepsy. The need for specific information on this patient group is likely to increase and future studies should address, among other things, the impact of AED treatment on quality of life, optimal choice of drugs, and impact of seizures on quality of life. Furthermore, qualitative studies on coping strategies and the impact of seizures on close relationships are of great interest. Pending these investigations, we hope that the data presented herein can be of use for clinicians counselling newly diagnosed patients, and for planning of future research.
